# A Reaction-Diffusion Model of Human Brain Development

**DOI:** 10.1371/journal.pcbi.1000749

**Published:** 2010-04-22

**Authors:** Julien Lefèvre, Jean-François Mangin

**Affiliations:** 1 LSIS, UMR CNRS 6168, Université Aix-Marseille II, Marseille, France; 2 LNAO, Neurospin, I2BM, CEA Saclay, Gif-sur-Yvette, France; University College London, United Kingdom

## Abstract

Cortical folding exhibits both reproducibility and variability in the geometry and topology of its patterns. These two properties are obviously the result of the brain development that goes through local cellular and molecular interactions which have important consequences on the global shape of the cortex. Hypotheses to explain the convoluted aspect of the brain are still intensively debated and do not focus necessarily on the variability of folds. Here we propose a phenomenological model based on reaction-diffusion mechanisms involving Turing morphogens that are responsible for the differential growth of two types of areas, sulci (bottom of folds) and gyri (top of folds). We use a finite element approach of our model that is able to compute the evolution of morphogens on any kind of surface and to deform it through an iterative process. Our model mimics the progressive folding of the cortical surface along foetal development. Moreover it reveals patterns of reproducibility when we look at several realizations of the model from a noisy initial condition. However this reproducibility must be tempered by the fact that a same fold engendered by the model can have different topological properties, in one or several parts. These two results on the reproducibility and variability of the model echo the sulcal roots theory that postulates the existence of anatomical entities around which the folding organizes itself. These sulcal roots would correspond to initial conditions in our model. Last but not least, the parameters of our model are able to produce different kinds of patterns that can be linked to developmental pathologies such as polymicrogyria and lissencephaly. The main significance of our model is that it proposes a first approach to the issue of reproducibility and variability of the cortical folding.

## Author Summary

The anatomical variability of the human brain folds remains an unclear and challenging issue. However it is clear that this variability is the product of the brain development. Several hypotheses coexist for explaining the rapid development of cortical sulci and it is of the highest interest that understanding their variability would improve the comparison of anatomical and functional data across cohorts of subjects. In this article we propose to extend a model of cortical folding based on interactions between growth factors that shape the cortical surface. First the originality of our approach lies in the fact that the surface on which these mechanisms take place is deformed iteratively and engenders geometric patterns that can be linked to cortical sulci. Secondly we show that some statistical properties of our model can reflect the reproducibility and the variability of sulcal structures. At the end we compare different patterns produced by the model to different pathologies of brain development.

## Introduction

The development of the human brain from the early gestational weeks to the buckling of the first folds at around 20 weeks follows a narrow pathway between determinism and pure randomness. On the one hand normal adult individuals offer quite similar - from a pure qualitative and descriptive point of view - folding structures: gyri and sulci. On the other hand we observe morphological variabilities between different brains [1]. This variability can reach extreme states in the case of rare abnormalities of the developing brain - such as lissencephaly, polymicrogyria or corpus callosum agenesis. The origin of variability remains an unclear and challenging issue [2] but it is, however, obvious that environmental factors have a deep impact on the sulcal and gyral pattern since even monozygotic twins exhibit important anatomical differences [3].

Folding or buckling are very general processes in nature and among living organisms. Especially one of the most studied step in the morphogenesis of metazoans is gastrulation which corresponds to a symmetry breaking of the spherical embryo and an invagination. The origin of this folding remains unknown even if mechanical factors are undoubtedly implied [4]. More disconcerting, it is shown in [4] that different mechanical actions (constriction, contraction, traction, gel swelling) can lead to similar shapes of the sea urchin primary gastrula.

In these conditions it raises the issue of realistic modeling of far more complex buckling processes such as the gyrification of mammal brains. In this regard it is important to inspect carrefully previous models of gyrification.

Le Gros Clark [5] raises first that the cortex grows by surface expansion rather than by increasing its thickness. He suggested that the expansion of the brain is constrained by the skull and basal ganglia and that compressive stresses cause sulcation. However experiments on sheeps whose large quantities of cortical and subcortical structures were ablated at the end of cellular migration revealed, at term, gyri and sulci of normal size and configuration [6]. This model and its refutation give us a way to categorize the hypotheses on the gyrification depending on whether they involve intra- or extra-cortical processes, in other terms intrinsic or extrinsic.

In the same extra-cortical point of view a recent and very popular model considers that the folding of the brain takes its origin in the mechanical tensions produced by the white matter fibers [7]. This model has been recently tested in [8] with a finite element model of cortical folding.

At the opposite there are numerous hypotheses arguing that the cortical folding has intrinsic origins. In [9] the differential growth of cortical layers causes sulcation and can explain anomalies of folding such as polymicrogyria and lissencephaly in terms of different mechanical properties of cortical tissues. Other models use mechanical hypotheses on the cortex such as elasticity or plasticity [10], [11]. In particular in [11] the authors suggest that the cortical folding is only a consequence of its growth modulated by anisotropies in mechanical or geometric properties. Even if this last model reproduces several characteristics of a growing cortex, it remains however implemented in 2D and does not explain completely where the anisotropies come from.

In the intrinsic origins of folding we encounter also purely morphogenetic hypotheses in which cortical convolutions are under genetic control [12]. In [13] it is proposed that the different cytoarchitectonic areas are provided by a protomap, that is a layer of predetermined neuronal units.

In the next part we will see in detail another hypothesis for the cortical folding which is based on Turing instabilities [14], [15]. We will show that these approaches of brain development can also be linked to the sulcal roots model proposed in [2]. These last authors offer indeed a descriptive model of the human sulcation based on the concept of sulcal roots that are elementary atoms around which the brain folding organizes itself. This concept has strong similarities with the sulcal pits one [16], [17]. Sulcal pits correspond to the deepest points of the sulci whose reproducibility has been demonstrated rigourously in [17]. We will see that initial conditions of the reaction diffusion process can have an interpretation in terms of sulcal roots or sulcal pits.

In this article we investigate the origin of anatomical variability from the early development and we propose a phenomenological model of the folding which is based on the putative existence of Turing morphogens. After recalling briefly some mathematical aspects of the model, we present the numerical schemes used for implementing the equations on a surface and for the deformation of the surface. We show some qualitative and quantitative results of the model. In particular we link sulcal pits maps to the average folding patterns across several realizations of a same noisy initial condition. And we study the variability of our model and demonstrate that it can lead to different modes of variability of one sulcus.

## Model

Cartwright [14] suggested first a similarity between labyrinthine Turing patterns and brain gyri. He proposed to model the cortical anatomy as obtained by a reaction-diffusion mechanism and more especially by Van Der Pol-Fitz Hugh Nagumo equations. The mechanism will correspond to axonal pathfinding with diffusing chemicals that activate axon growth or inhibit it [18].

Very recently the Global Intermediate Progenitor (GIP) model has been proposed to explain the appearance of transversal or sectorial sulci following the Intermediate Progenitor hypothesis [19]. The GIP model is based on the BVM system of reaction-diffusion equations [20] that mimics the patterning of the subventricular zone.

We aim at extending the analogy first formulated by Cartwright and the GIP model using a system of reaction-diffusion equations that will modify the surface on which the equations take place. Namely the reaction diffusion system models the non-linear interaction of two morphogens 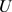 and 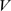 or in other terms growth factors. These growth factors are characterized by the fact that they will tend to deform the surface on which they evolve. We can note that this approach has been previously used in the case of plant growth [21] [22] and that reaction-diffusion equations on surfaces have also been adopted to apply textures on meshes [23] [24].

In our model initial conditions of the reaction-diffusion equations have strong similarities with the sulcal roots described in [2], that are the initial seeds of the folding process.

Our choice of the reaction-diffusion equations differs from the one of Cartwright and the GIP model since we have adopted the Gray-Scott model:
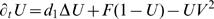
(1)

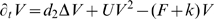
(2)which exhibits a high number of patterns for differents values of 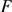 and 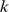 [25].

The mathematical analysis of the model has been previously conducted in [26]. As in [15] we have adopted a phenomenological approach and it is interesting to note that our model and the BVM one differ only by a quadratic term. Linear and cubic terms in the kinetic reactions are present in the two models.

### Surface deformation

Following ideas of [21] and [22], we suppose that the evolution of the studied surface 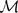 is driven by the morphogens 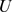 and 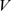. 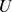 is the inhibitor and 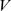 the activator. In mathematical terms we have that
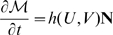
(3)where 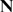 is the normal to the surface and 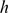 a function of the two morphogens. The simplest case for 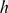 that we have adopted in the following is a linear function of one morphogen:
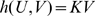
where 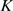 is a parameter in 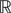.

Since the surface on which evolve the morphogens is modified with time, we have to adapt the equations (1) and (2) to take into account the geometric changes. The problem of reaction-diffusion on growing domains has been well-studied in the past years. It leads generally to add convective and dilution terms to 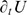 (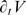 respectively) that can be combined in 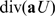 where 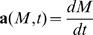 represents the flow velocity of the growing surface [27]. However this result does not directly apply to surfaces and we have to refer to [28] to see the influence on the curvature changes on the reaction-diffusion equations.

The model proposed in [28] consists in adding a term reflecting the modification of the surface metric along time. If the surface 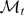 is parameterized by 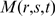 then equations (1) and (2) read:
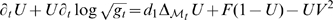
(4)

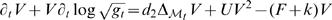
(5)where 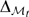 stands for the Laplace-Beltrami operator. 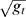 is the determinant of the metric 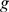 associated to the surface, that is:
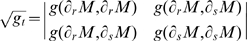
In the following we will use 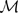 instead of 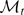 and 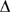 instead of 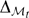 for simplicity reasons but one has to remember that the surface on which the equations are defined is changing along time.

### Numerical implementation

Since we work on discrete meshes we have used a finite element method to discretize the linear terms in the equations 1 and 2.

First we derive a weak formulation of the system on
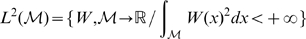
with non-linear terms included in 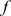 using a test function 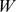:
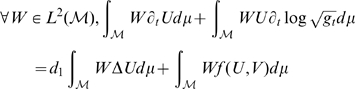
Then, integrating by part the Laplacian term, as in [29], we get:
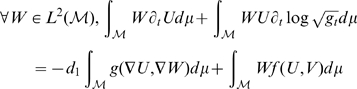
where 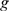 is the metric associated to the Riemannian manifold 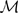.

Next we work on a discrete tessellation 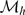 of the surface 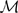 composed of 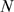 vertices. We define 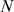 functions 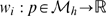 which are continuous piecewise affine, with the property to be equal to 1 at node 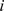 and 0 at all other triangle nodes. They are the basis functions for the approximation on the functional space of finite dimension 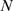. So any function 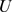 continuous piecewise affine reads: 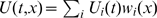.

The weak formulation becomes:
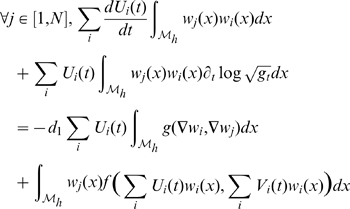



It is possible to treat the non-linear term with the following approximation as in [30]:
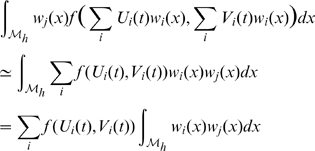
then discretizing implicitly and explicitly in time between 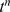 and 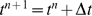 and writing with matricial expressions:
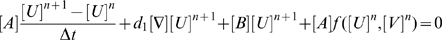
with
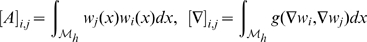


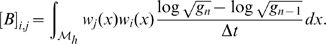
and by definition
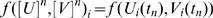
So we can deduce:
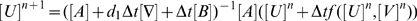
On each triangle we have
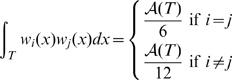


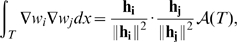
where 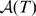 is the area of triangle 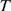 and 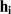 is the height of triangle 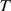 from vertex 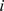.

At last we need to compute 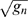. This can be performed on each triangle 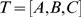 from the expression of the metric tensor 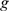 [31]:
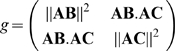



#### Remarks

To be exact we should take into account the fact that the finite elements vary along time or in other terms that the mass matrix 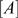 depends on the evolving surface. This point is addressed in [32] but it is not possible to apply this framework in our case since we should know the geometry of the mesh at time 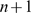 to compute the mass matrix 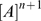 which is not the case because the mesh at time 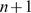 is deduced from 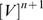. However the authors in [32] propose an equivalent weak formulation in which the velocity term is present. In our case the dilution term replaces the velocity term. Moreover the expression of 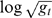 should involve 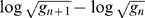 but for the same reason as previously we have not the knowledge of 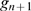 at time n.

**Figure 1:**
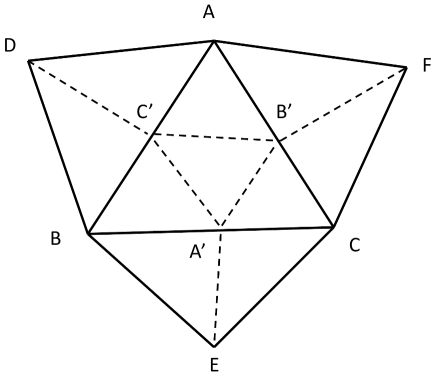
Mesh refinement procedure. When the area of a triangle ABC exceeds two times the averaged area of the triangles of the original mesh we divide the original triangle in four triangles constructed from the three midpoints A′,B′,C′ of each side. Moreover we divide each of the three triangles ABD, ACF, BCE in two triangles.

For the surface deformation step, we translate equation (3) by simply modifying each vertex 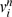:
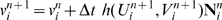
(6)Although this incremental procedure can rapidly lead to abnormal deformation of the original mesh, we have used triangle refinements in order to prevent this issue. When the area of a triangle exceeds two times the averaged area of the triangles of the original mesh we simply divide the triangle in four triangles constructed from the three midpoints of each side Fig. 1.

In our implementation we do not prohibit self-intersection which would increase considerably the computation time. However we can say that we escape this issue by not solving for too long time but also taking a parameter 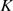 for the deformation along the normal not too large. By this we mimic also the real brain expansion that is not confronted to the problem of gyri collision.

## Results

### Labyrinthine patterns

**Figure 2:**
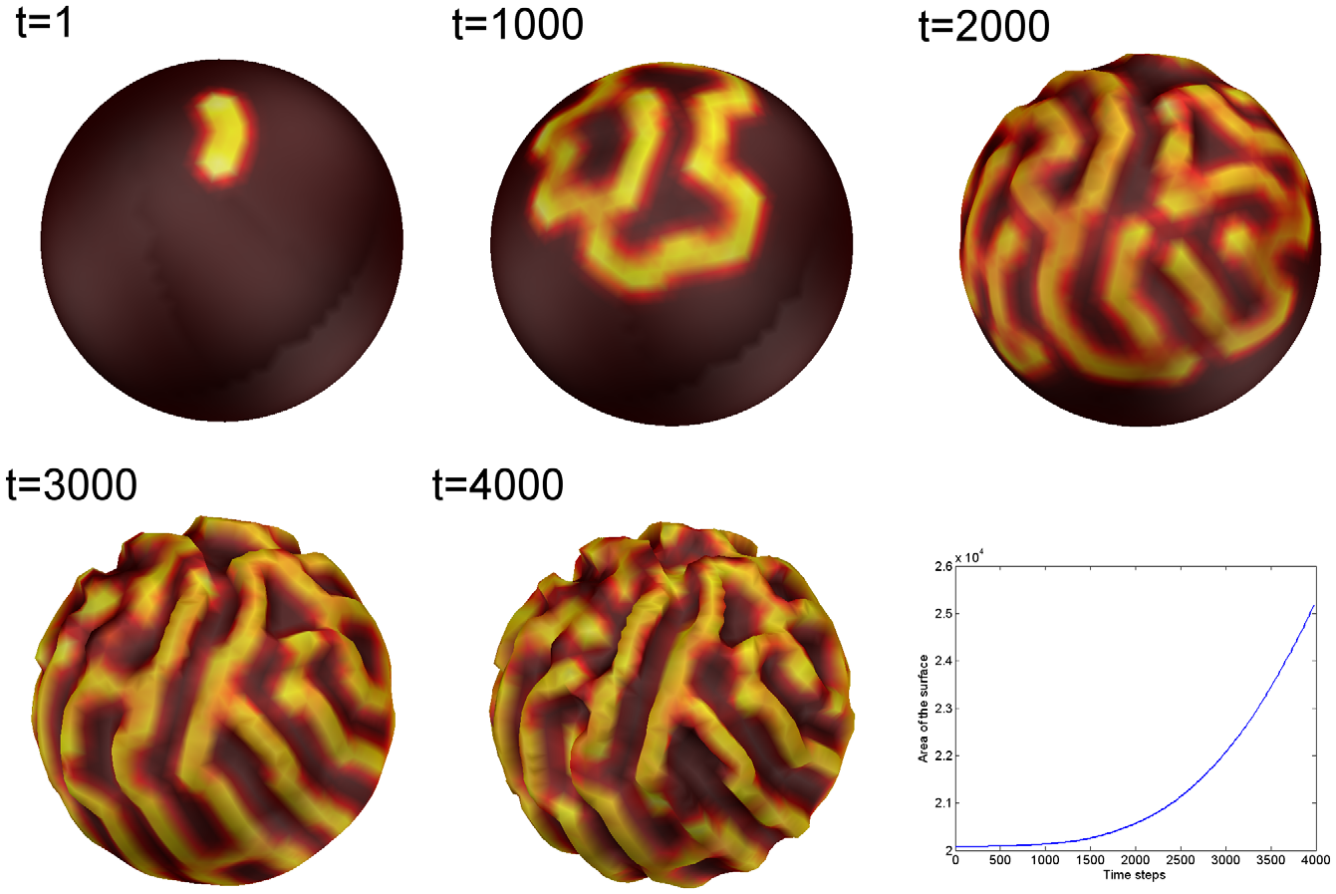
Evolution of the Gray-Scott model coupled to surface deformation. The pictures correspond to time instants 1, 1000, 2000, 3000, 4000 of the iteration process. The last graph indicates the evolution of surface area in the successive meshes.

First we can model the growth of a normal brain with the value 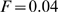, 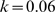, 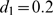, 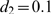, 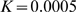 and 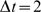. The initialization corresponds to a slight perturbation of the stable equilibrium 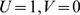 in a position of a sphere composed of 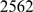 vertices. This perturbation consists of a broad line with 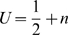 and 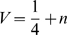 where 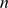 is white noise of amplitude 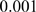 (see first picture on Fig. 2).

**Figure 3:**
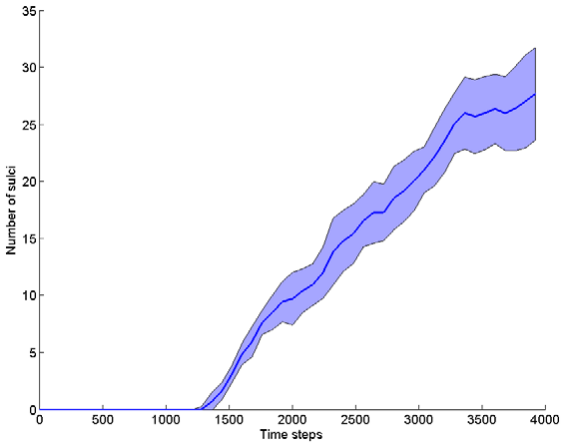
Evolution of the number of folds along time in 50 simulations. The blue line represents the average number across 50 simulations and the blue area around represents the standard deviation.

Note that the evolution specified by the coupled reaction-diffusion equations and the surface deformation leads to a progressive folding of the initial sphere on Fig. 2. It is possible to extract an order parameter at each time step which consists of the number of folds or sulci (see Fig. 3 left). This index is defined from the curvature map of the surface. At each vertex of the mesh we compute the mean curvature following [33]. Once this curvature map has been obtained we compute automatically the number of sulci, that is, the number of connected components whose curvature is inferior to 0. For this we use a region growing algorithm: we start from a vertex whose curvature is inferior to 0 and build a connected region of vertices whose curvature is inferior to 0. We repeat this procedure until there are no more initial seeds.

We can observe on Fig. 3 that the number of sulci is equal to 0 on the interval 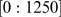 then increases quasi linearly on 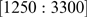 and reaches a sort of plateau on 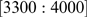.

**Figure 4:**
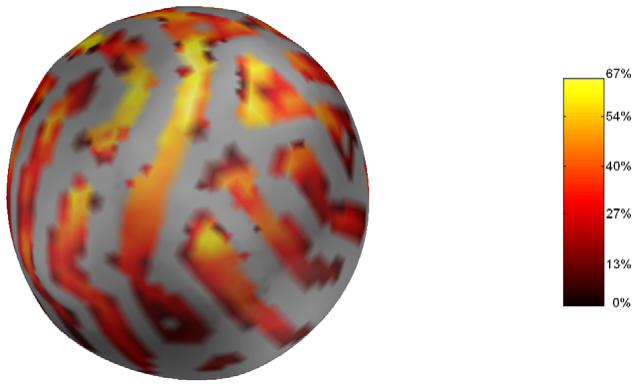
Stability of folds in time. Time-average map 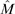 representing the proportion of the temporal interval during which a fold is present at position 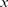. The values goes from 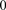 (gray) to 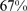 (yellow).

Moreover we propose a simple way to characterize the spatial stability of the folds along time. In other terms we demonstrate that the position of folds formed at different time instants remains relatively stable. We extract a map of the curvature 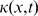 at each time instant 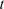. And we define a thresholded map 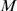 by 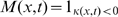. This map depends on the mesh on which it is defined so we interpolate it on the final mesh (which has the largest number of vertices). We note that we use a smoothed version of the meshes in order to avoid problems of interpolation. The smoothing of the folded meshes has been performed by using an iterative process that consists, at each iteration, to replace a node of the mesh by the mean of its neighbors. So the maps 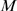 are defined on the same domain and we can compute an average map
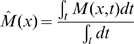
Intuitively this quantity represents the proportion of the temporal interval during which a fold is present at each position 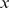. This yields the map shown on Fig. 4.

We can see on this figure that the average map 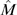 is not uniform but has patterns. In other terms we can observe a certain stability of the folds along time. In particular there are parts of the initial sphere that never belong to a fold. We note that the maximum of 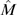 is not 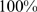 since there are no folds during the temporal interval 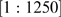 which represent 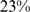 of the full temporal interval on which the simulation has been performed.

### Influence of noise

**Figure 5:**
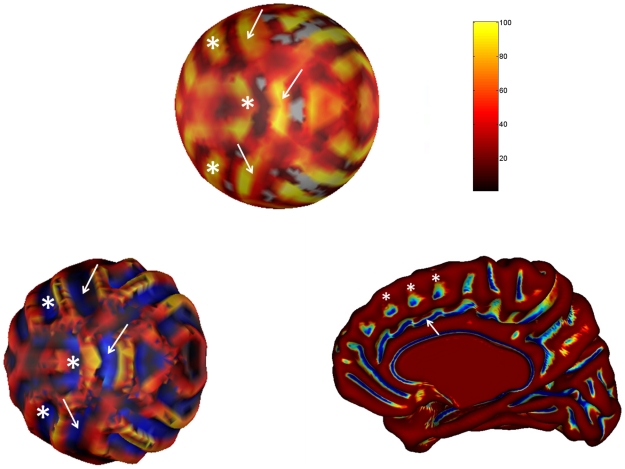
Reproducibility of the model compared to real data. Top: Sum of the thresholded maps 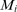 converted in percentage. Areas of higher percentage (yellow) correspond to domains of high reproducibility of the folding patterns. The white arrows indicate the three parts of the main fold. Bottom left: Average surface of 50 simulations taken at time t = 4000 and the corresponding curvature in color. The white arrows indicate the three parts of the main fold. Bottom right: Average cortical surface of 222 hemispheres taken from experimental data. The white arrow indicates the cingulate sulcus while the four white arrows show secondary folds (paracingulate sulcus). In these three figures the white stars indicate the position of secondary folds.

In this part we investigate the influence of noise in the spatial position of the folds. In particular we aim at demonstrating that the reaction diffusion mechanism is able to produce reproducible folds at certain specific locations but can also engender variability at other locations. For this we simulate 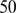 realizations of the folding process from different noisy initial conditions 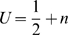 and 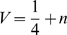. We consider the curvature maps 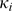 and the thresholded maps 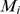 at time 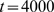 and interpolate them on the same smoothed mesh. Then we sum the binary thresholded maps in order to see areas of reproducibility:
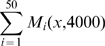
On Fig. 5 left, we can clearly observe that the sum of the binary maps representing the averaged pattern of folding have a spatial structure and do not organize randomly. In particular we notice a big longitudinal fold that comes across the surface and seems to be very reproducible among the 50 simulations. Moreover we note that other smaller reproducible folds are positioned along the main fold on both sides. This figure echoes the average cortical surface of 222 hemispheres that we have displayed on Fig. 5 right. This surface is the one described in [34] and has been visualized with anatomist [35]. The white arrow represents the cingulate sulcus that is comparable to our main fold in the 50 simulations while the three white stars show secondary folds that are parts of the para-cingulate sulcus and that we can link to the smaller reproducible folds of our model.

**Figure 6:**
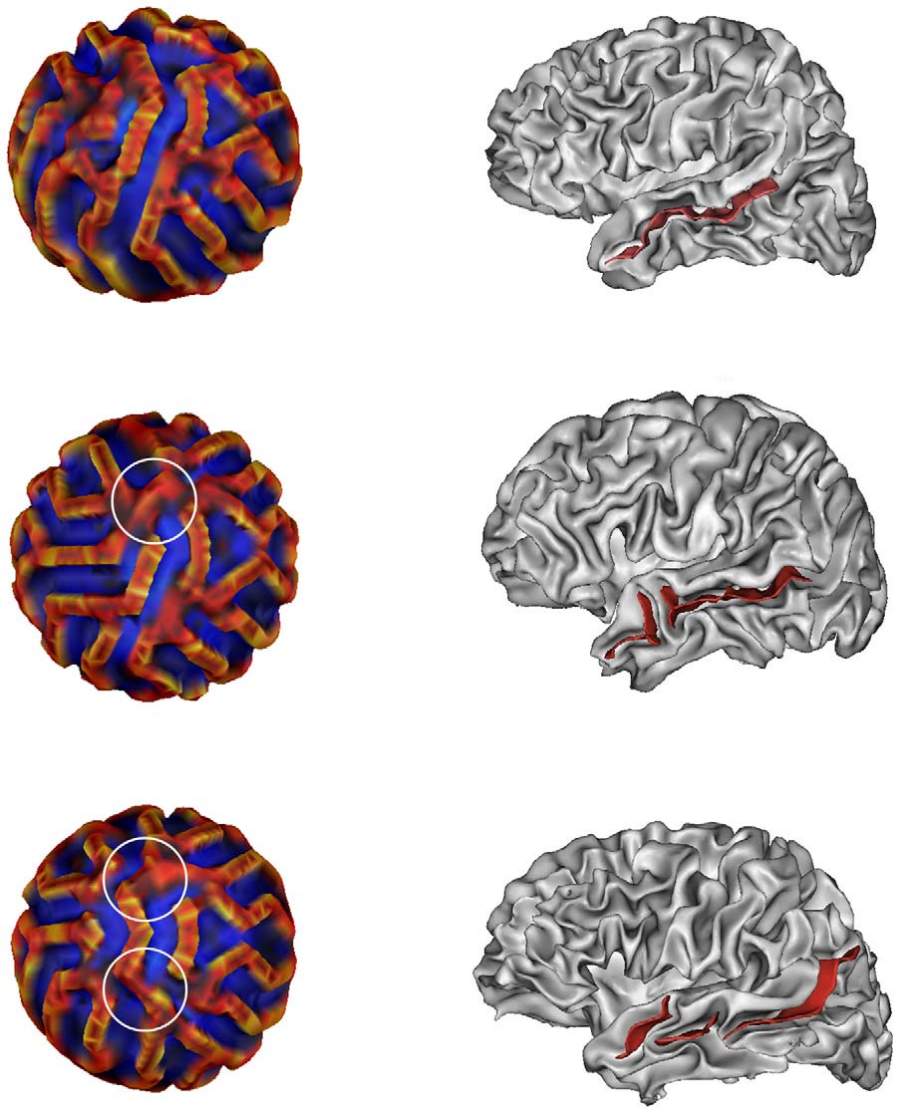
Three modes of variability and their correspondence on real anatomies. First column: Three different modes of variability for the main fold observed on Fig. 5. We can see that the main sulcus, in one part at top left, is interrupted by a gyrus surrounded in white at middle left, and interrupted by two gyrus at bottom left. Second column: Two different modes of variability for the superior temporal sulcus on experimental data. Top: the superior temporal sulcus (STS) in pink is in one part. Middle: the STS is in two parts. Bottom: the STS is in three parts.

On Fig. 6 we illustrate however the variability of the main fold through three different scenarios of buckling. On the first line, left figure, we can see a mode that follows the main distribution previously described on Fig. 5 that is, in which the main fold is in one part. On the right is shown a left hemisphere of a real brain displayed with anatomist [35] on which the superior temporal sulcus (pink) is in one part. On the two following lines we represent two other modes for the main fold, in two and three parts respectively, and their correspondence on real anatomies with a superior temporal sulcus (pink) in two and three parts respectively.

**Figure 7:**
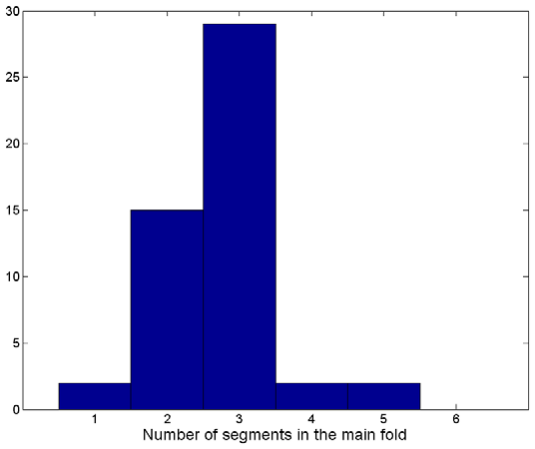
Different modes of the main fold.

More generally Fig. 7 shows the different modes of variability of the main fold among the 50 simulations. In 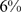 of cases it is composed of one segment, in 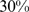 of two segments, in 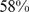 of three segments and in 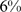 of four and five segments. The determination of connected components has been done by visual inspection rather than by automatic methods that tend to increase artificially the number of segments in the main fold.

### Phase diagram and pathologies of gyrification

**Figure 8:**
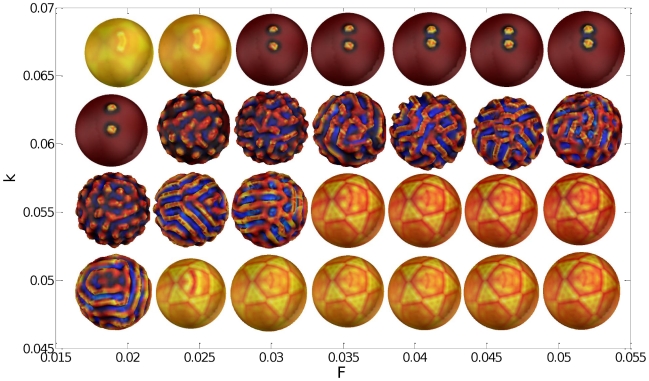
Phase diagram of the Gray-Scott model. The two axes correspond to the two parameters 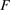 and 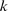 used in the Gray-Scott model. The color indicates the curvature of the surfaces (red: positive curvature, blue: negative curvature) obtained at time 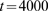.

It is possible to represent directly the influence of one or several parameters of the model (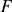 and 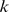 in our case) on the qualitative properties of the patterns. We vary the parameters 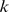 and 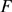 linearly over a spatial domain from 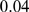 to 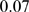 and 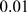 to 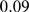 respectively with steps of 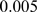 which yields 28 couples 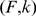. On Fig. 8 we display the surfaces obtained at time 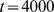. The color represents the curvature of these surfaces (red: positive curvature, blue: negative curvature). Note that the star-like patterns obtained with values 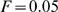, 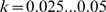 and 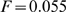, 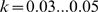 are just an artifact corresponding to the structure of the spherical mesh.

**Figure 9:**
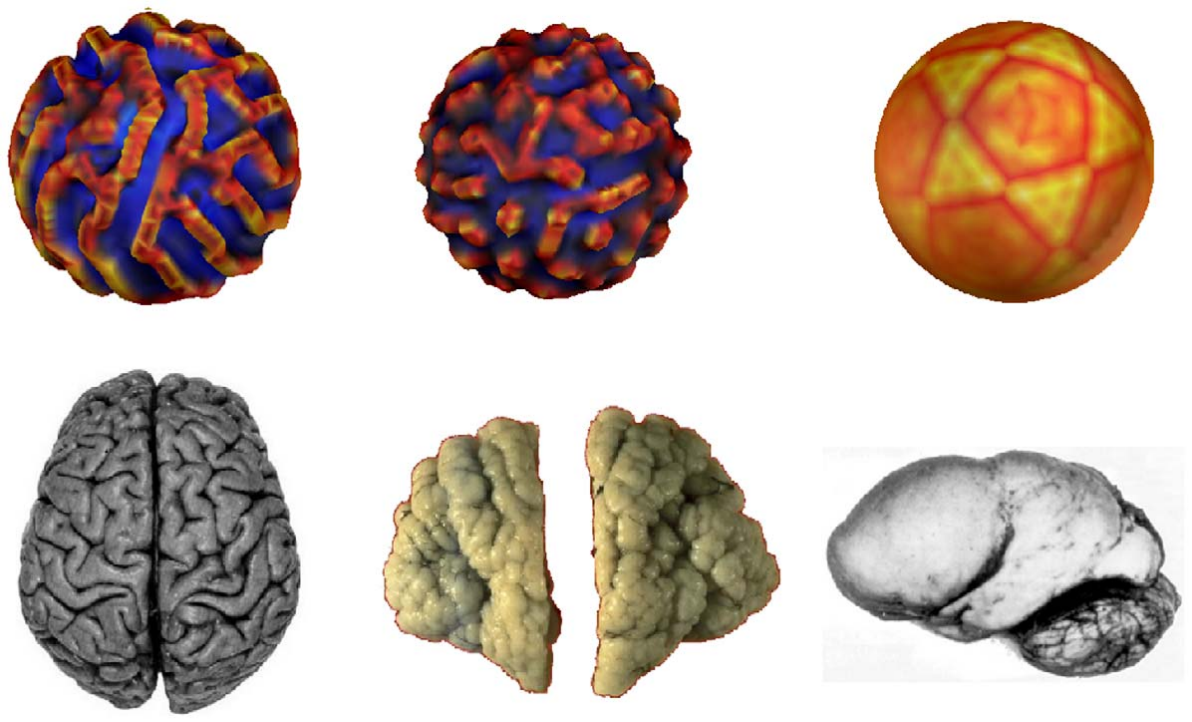
Patterns of folding and pathologies of development. First line: Growth patterns for different values of (F,k), respectively (0.04,0.06), (0.03,0.06) and (0.05,0.05). Second line: the corresponding brain patterns: normal, polymicrogyria, lissencephaly.

As suggested by [14] it is possible to link the qualitative nature of the obtained patterns to different modes of brain development, i.e in particular to pathologies or anomalies (see Fig. 9).

So we can see that for 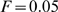 and 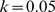 no patterns emerge. This state appears similar to lissencephaly, a pathology in which the brain is smooth and offers no gyri or sulci. The values 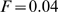 and 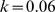 might correspond to a normal brain with stripe-like patterns of gyrification. At last the range 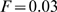 and 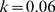 show spot-like patterns which make one think of polymicrogyria.

## Discussion

Our model extends the initial proposal of Cartwright in [14] where no geometrical deformation of the cortical surface was proposed. We have demonstrated that it was possible to combine a reaction diffusion mechanism to a surface deformation in order to produce a model of the gyrification process. This approach is not new since it has been applied to model plant growth [21] but it seems to be the first to tackle the very old and controversial problem of brain folding in terms of reaction diffusion coupled to surface deformation. However the question about the origin of the morphogens used in our model remains open. In [14] the activation/inhibition process is supposed to model the mechanical tensions due to white matter fibers so the morphogenetic approach becomes indirect and extrinsic. On the contrary we prefer to view the folding process as the result of an intrinsic phenomenon, promoted by morphogens that decide the cytoarchitechtony. Different cytoarchitechtonic areas would correspond to different gyri and the limits between areas to sulci. This idea, suggested one century ago by Broadmann, has been recently pointed out in [36]. Moreover in [15], the GIP model supposes that the morphogens responsible for the patterning of subventricular zone could be some specific genes such as *Pax6*, *Ngn2*, *Id4*. Our model supports this hypothesis since mutations in the *Pax6* gene for instance can be responsible for polymicrogyria [37], so the parameters 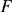 and 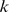 of the model could reflect different gene expression of *Pax6*. We can also mention an alternative scenario for pattern formation that has been recently exposed in [38] and does not necessarily require the interaction of a long range inhibitor (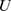) and a short range activator (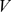) as in our case. In [38] an activator-activator mechanism combined with domain growth can also lead to pattern formation.

In our model we investigate also the variability of folding along the development of one individual and across several individuals - that is several realizations of the model. First we can see that for an unique development the position of the sulci remains stable along time. This result may seem trivial but is required for our model to produce definite patterns of gyrification that can be compared between different realizations of the model. Secondly the study of folding variability among 50 random realizations of the model reveals two important characteristics. The folding does not organize randomly even if we add noise to the initial condition of the reaction diffusion process. We have shown on one example that a main structure emerges that is strongly reproducible among several simulations. We can find a direct analogy between this main fold and the primary folds described in the literature [1], [39]. Primary folds are indeed characterized by their early time of appearance and their reproducibility across subjects. If we follow the comparison we can link the smaller structures found on Fig. 5 to the secondary or tertiary folds that are more posterior and variable than the primary ones. Our average map on Fig. 5 left echoes the average cortical surface of 222 hemispheres displayed on Fig. 5 right. This average surface has been computed in [34] and used also to represent an average map of sulcal pits density in [17]. In particular the main fold found on our simulations can be compared to the cingulate sulcus while the smaller folds around evoke the small pits of the paracingulate sulcus.

Moreover we have shown that in spite of its strong reproducibility the main fold could be broken in two separate parts by a gyrus. This result echoes previous studies [39], [40] where it is shown that some primary sulci reveal variability in their topology. For instance in [40] the superior temporal sulcus is continuous in one third of the cases (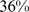 on the left and 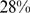 on the right), in two segments in 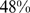 on the right and 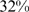 on the left. The gyri that separate our main structure in two or three parts could also be interpreted in terms of ‘pli de passage’, which is a fold that can divide a sulcus in two sulci or just be buried at the bottom of a sulcus [2].

On a more theoretical point of view, our results on the reproducibility of the folds seem to confirm the impact of growth domain on the robust selection of patterns as it has been previously shown in [41] [42]. In our study there remains however some points that will require some theoretical developments, in particular about the existence of Turing instabilities that occur in the simulations. Some results have been obtained recently for isotropic domain growth or specific growth function [43] [38].

In conclusion we have proposed an extended framework for modelling the cortical folding. It is based on a system of coupled reaction-diffusion equations defined on a surface that evolves through the action of morphogens. We show that for some parameters the model gives rise to geometric patterns that can be related to cortical sulci. We also demonstrate that under the effect of noise the system yields morphological variability in these cortical structures. Moreover changing slightly the values of the parameters of the model can have an important influence on the nature of the created patterns which suggest a link toward pathologies of the brain development such as lissencephaly or polymicrogyria. In future developments we plan to investigate the difficult issue of estimating good values of parameters with respect to a given sequence of cortical surfaces across development.
